# *Blumea chishangensis* sp. nov. (Asteraceae: Inuleae) from Taiwan and new insights into the phylogeny of *Blumea*

**DOI:** 10.1186/s40529-022-00350-z

**Published:** 2022-07-12

**Authors:** Shih-Wen Chung, Wei-Jie Huang, Zhi-Hao Chen, Shih-Hui Liu

**Affiliations:** 1 Botanical Garden Division, Taiwan Forestry Research Institute, Taipei, 100 Taiwan; 2 Forestry Research Institute Herbarium (TAIF), Taiwan Forestry Research Institute, Taipei, 100 Taiwan; 3 Observer Ecological Consultant Co., Ltd., Taipei, 103 Taiwan; 4 Department of Biological Sciences, National Sun Yat-sen University, Kaohsiung, 804 Taiwan

**Keywords:** *Blumea chishangensis*, Compositae, Taiwan, Taxonomy, Molecular phylogeny

## Abstract

**Background:**

*Blumea* plants are widely distributed in the tropical areas of Asia, Africa, and Australia, especially tropical Asia. Limited studies left the taxonomy and infrageneric phylogeny of *Blumea* insubstantial. Here, a new species, *Blumea chishangensis* S. W. Chung, Z. H. Chen, S. H. Liu & W. J. Huang, from Taiwan is described, and an extended phylogeny is reconstructed to provide new perceptions of *Blumea* evolution*.*

**Results:**

The new species is distinguished from *B. hieraciifolia* by the following features: leaf blade sparsely pilose or glabrescent (vs. silky villous), the leaves margins regularly remote mucronulate (vs. double serrate or dentate), capitula pedicelled (vs. capitula sessile or subsessile), and leaves basal rosette or sub-basal rosette and a few cauline (vs. mostly cauline). Phylogenetic analysis based on the ITS, *trnL-trnF*, and *trnH-psbA* regions places the new species in the subclade II in *B. lacera* clade and shows a close relationship with *B. axillaris* and *B. oxyodonta*. A key to *Blumea* species in Taiwan and the studied species in the subclade II is provided. Moreover, the evolutionary inferences of *B. conspicua*, *B. linearis*, and *B. sinuata* are first reported here. The paraphyly of *B. formosana* and *B. sinuata* are also revealed for the first time.

**Conclusions:**

Both morphological and molecular data support that *B. chishangensis* is a new species. Our phylogeny highlights the need for further taxonomic and evolutionary studies on *Blumea.*

**Supplementary Information:**

The online version contains supplementary material available at 10.1186/s40529-022-00350-z.

## Background

*Blumea* DC. is one of the largest genera in the Tribe Inuleae, comprising approximately 100 species (Randeria 1960; Anderberg and Eldenäs 2007; Pornpongrungrueng et al. 2016) distributed in tropical Asia, Africa, and Australia, with the highest diversity occurring in tropical Asia (Pornpongrungrueng et al. 2016). *Blumea* is characterized by its disciform capitula, presence of one large oxalate crystal in each cell of the cypsela epidermis, radial anther endothecium thickenings, and tailed anthers. Despite being common in southeast Asia, the taxonomy of *Blumea* has not been thoroughly studied (Randeria 1960; Grierson 1972; Koyama 1984). The number of taxonomic revisions regarding this genus is still limited, with only a partial revision of 49 species of *Blumea* worldwide by Randeria (1960) and another revision of 27 species in continental southeast Asia by Pornpongrungrueng et al. (2016). In addition to the lack of taxonomic studies, the inconsistent and indistinct traits among some species and the heterogeneity of the genus increase the difficulty of determining the boundaries between different species (Pornpongrungrueng et al. 2016).

Previous phylogenetic studies had revealed that *Blumea* is a sister group of the genus *Caesulia* Roxb. (Anderberg et al. 2005; Pornpongrungrueng et al. 2007; Zhang et al. 2019). Combining evolutionary information of the ITS, *trnL-trnF*, and *trnH-psbA* regions from 30 *Blumea* species, Pornpongrungrueng et al. (2007, 2009) were the first to examine the infrageneric relationships of *Blumea* and identified three clades in the genus––*B. lacera* clade, *B. densiflora* clade, and *B. balsamifera* clade. Their phylogeny showed that the relationships among the three clades were unresolved and pointed out that several taxa were not monophyletic. Zhang et al. (2019) investigated the infrageneric relationships of 22 Chinese *Blumea* taxa with the ITS and *trnL-trnF* regions, recognized two clades. The clade I, which contained *B. densiflora* clade and *B. balsamifera* clade recognized by Pornpongrungrueng et al. (2007, 2009), however, had low supports in Zhang et al. (2019) phylogenies. The clade II was equal to *B. lacera* clade in Pornpongrungrueng et al. (2007, 2009). Within clade II (hereafter call *B. lacera* clade), Zhang et al. (2019) identified three subclades and two single species.

**Fig. 1:**
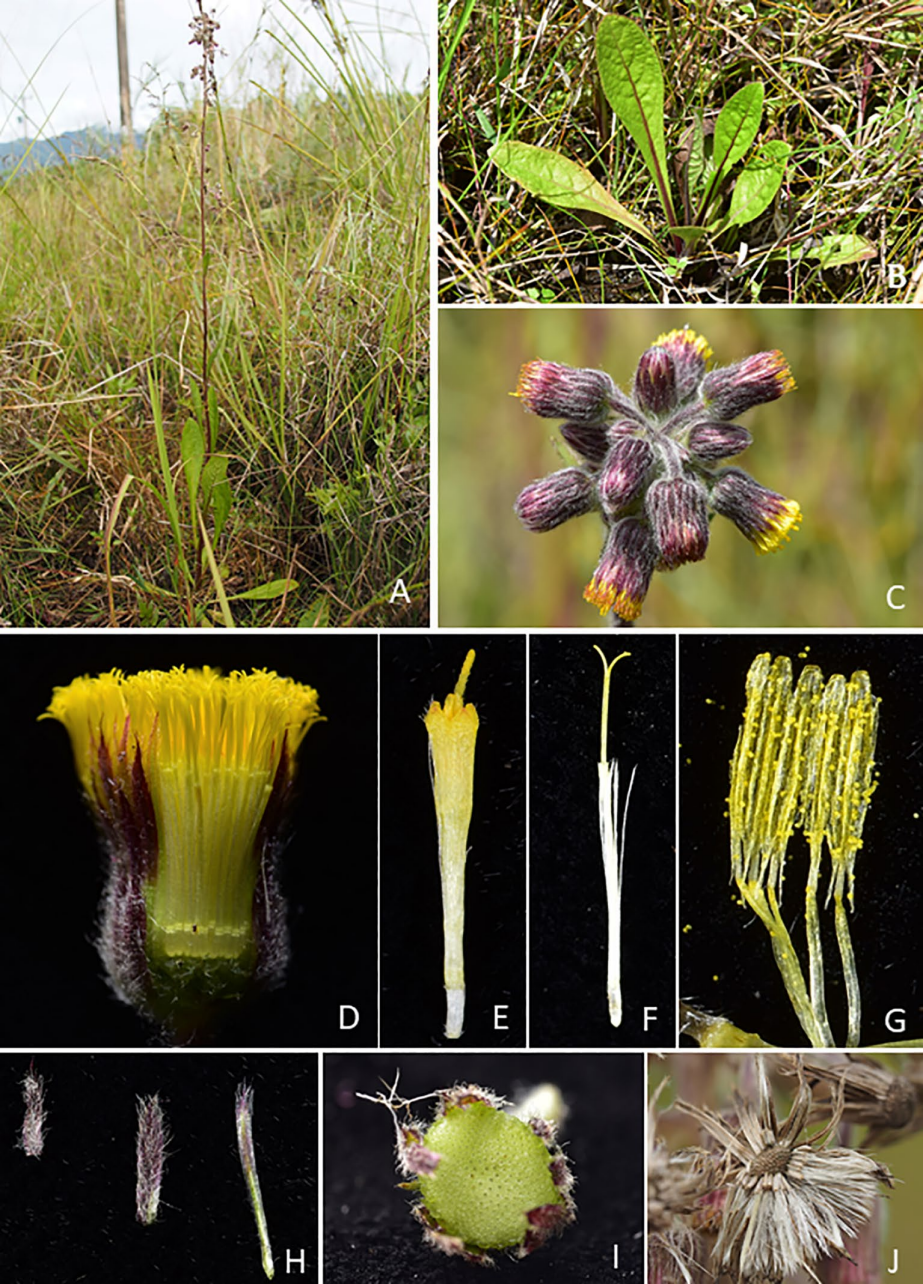
*Blumea chishangensis*. **A** habit; **B** basal leaves; **C** portion of inflorescence; **D** dissected capitula; **E** central floret; **F** outer floret; **G** stamens; **H** involucral bracts; **I** receptacle; **J** receptacle and achene

**Fig. 2:**
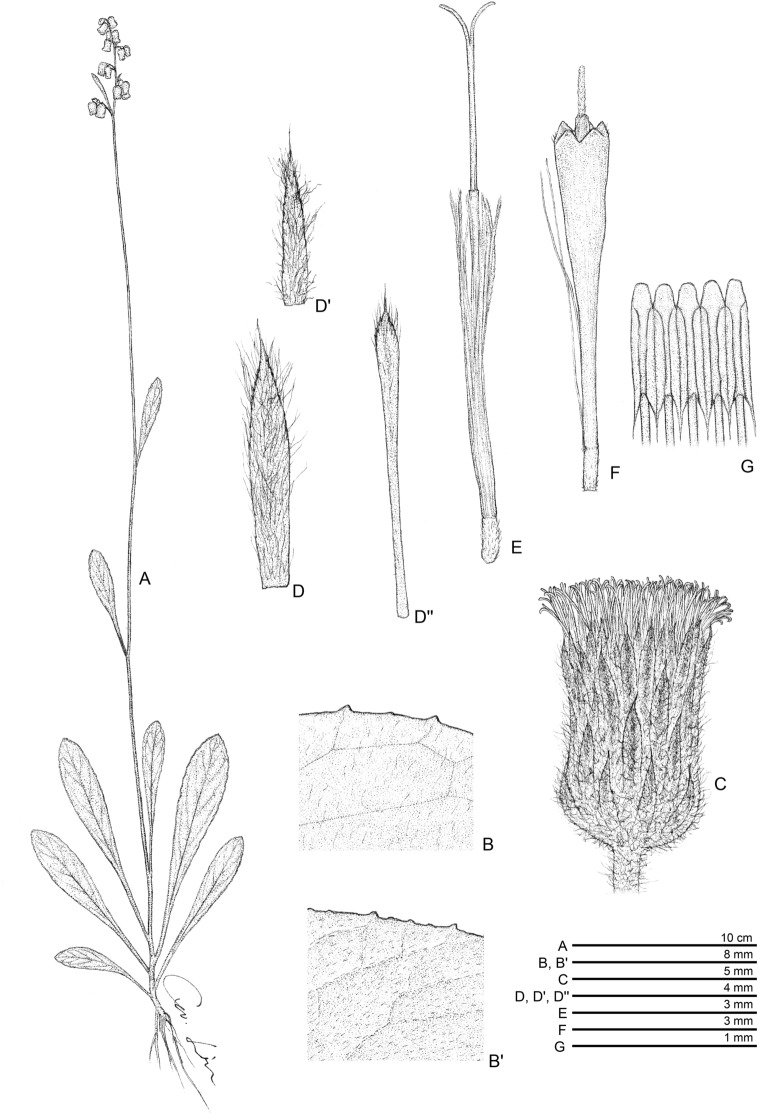
*Blumea chishangensis*. **A** habit; **B** basal leaves, upper surface; **B'** basal leaves, lower surface; **C** head; **D**, **D"** involucral bracts: **E** outer floret; **F** central floret; **G** stamens

Twelve species of *Blumea* were recorded in Flora of Taiwan, including *B. aromatica*, *B. balsamifera*, *B. conspicua*, *B. formosana*, *B. hieracifolia*, *B. lacera*, *B. laciniate*, *B. lanceolaria*, *B. axillaris*, *B. oblongifolia*, *B. riparia* var. *megacephala* and one endemic species, *B. linearis* (Leu and Peng 1998). During the fieldwork in the southeast of Taiwan in 2019, the authors found a *Blumea* population from Chishang Township which did not match any of the known *Blumea* species (Henry 1896; Randeria 1960; Leu and Peng 1998; Chen and Anderberg 2011; Pornpongrungrueng et al. 2016). After careful examination of relevant literature and specimens in Taiwanese herbaria, we conclude that it is an undescribed species (Figs. 1, 2). Hence, it is proposed as a new species with detailed morphological descriptions. A key to *Blumea* in Taiwan is also provided.

To understand the evolutionary position of the new species as well as to give new insights into the infrageneric relationships of *Blumea* with additional taxa and molecular data, phylogenetic analysis based on nuclear ITS and two plastid DNA sequences was conducted in this study.

## Methods

### Plant materials and morphological investigation

For morphological comparison, the living materials of all 12 Taiwanese *Blumea* species (Leu and Peng 1998) and *Blumea* specimens preserved in HAST, TAI, and TAIF were carefully observed, photographed, and measured. For taxa whose materials are not available in Taiwan, their morphological data were adopted from literature, chiefly the latest monographic work of Pornpongrungrueng et al. (2016). Digital specimen images available in the online databases of IBSC, IBK, K, KUN, P, and PE were also consulted for reference.

For molecular phylogenetic analyses, the new species and the majority of Taiwanese *Blumea* taxa were collected from the field and planted in the research greenhouse of Taipei Botanical Garden. The fresh leaf materials for the following molecular analyses were dried in silica gel immediately right after collection. The vouchers applied for our morphological investigation were deposited in the TAIF herbarium for further studies.

### Genomic DNA isolation, PCR, and sequencing

Genomic DNA of the newly collected *Blumea* young leaf materials was isolated with the Plant Genomic DNA Extraction Miniprep System (Viogene, Taipei, Taiwan) and kept at − 20 °C before the amplifications. Following the earlier studies focusing on *Blumea* (Anderberg et al. 2005; Pornpongrungrueng et al. 2007, 2009; Zhang et al. 2019; Abdullah et al. 2021), both nuclear ITS (ITS1 + 5.8S + ITS2) and chloroplast (*trnL-trnF* and *trnH-psbA* spacers) regions were used in the present study to infer the phylogenetic positions of the described Taiwanese *Blumea* taxa and the new species. With seven sets of primers (Table 1), all three regions were successfully amplified for all newly collected *Blumea* samples. The primer sequences, sources, optimal conditions for the PCR reactions, and PCR product sizes of the studied DNA regions are shown in Table 1. Each PCR mixture contained 1 μL genomic DNA, 1 μL 10 μM forward primer, 1 μL 10 μM reverse primer, 5 μL 5 × FIREPol Master Mix (Solis BioDyne, Tartu, Estonia), and 17 μL nuclease-free water. The PCR product purifications and DNA sequencing in an ABI 3730xl DNA Analyzer were then conducted commercially with the services at the Genomics BioSci & Tech. Co., Ltd. New Taipei City, Taiwan.

**Table 1:** The PCR primers, optimal annealing temperatures, and product sizes of ITS, *trnL-trnF*, and *trnH-psbA* regions in this study

DNA regions	Primers	Primer Sequences (5′–> 3′)	Primer sources	Annealing temperature (°C)	Product size (bp)
Nuclear region					
ITS (ITS1 + 5.8S + ITS2)	ITS_4 ITS_5	TCCTCCGCTTATTGATATGC GGAAGTAAAAGTCGTAACAAGG	White et al. 1990 White et al. 1990	55	662–666
	ITS_L ITS_Scleria	TCGTAACAAGGTTTCCGTAGGTG ATGCTTAAACTCAGCGGGTA	Hsiao et al. 1994 Liu et al. 2021	60	662–666
Chloroplast regions					
*trnL-trnF*	trnLF_c trnLF_f	CGAAATCGGTAGACGCTACG ATTTGAACTGGTGACACGAG	Taberlet et al. 1991 Taberlet et al. 1991	60	829–895
	trnLF_c trnLF_d	CGAAATCGGTAGACGCTACG GGGGATAGAGGGACTTGAAC	Taberlet et al. 1991 Taberlet et al. 1991	60	466–471
	trnLF_f trnLF_e	ATTTGAACTGGTGACACGAG GGTTCAAGTCCCTCTATCCC	Taberlet et al. 1991 Taberlet et al. 1991	59	369–383
*trnH-psbA*	psbAF trnH2	GTTATGCATGAACGTAATGCTC CGCGCATGGTGGATTCACAATCC	Sang et al. 1997 Tate and Simpson 2003	64	460–494
	trnH2 psbABL	CGCGCATGGTGGATTCACAATCC AGCTGCTTGGCCTGTAGTAG	Tate and Simpson 2003 This study	63	505–637

To understand the phylogenetic positions of Taiwanese *Blumea* taxa, additional 101 sequences from 26 *Blumea* taxa and 12 sequences from four taxa in the close relative genera (*Aster* L., *Caesulia*, *Laggera* Sch. Bip. ex Benth. & Hook. f., and *Pluchea* Cass.) were downloaded from the NCBI. The GenBank accession numbers of the studied sequences and sampled taxa are available in Additional file 1.

### Sequence assembly, alignment, and phylogenetic analyses

Sequence reads were trimmed and assembled using both the De Novo Assemble and Map to Reference tools implemented in Geneious Prime 2022.1 (https://www.geneious.com) with the default settings. The ITS, *trnL-trnF*, and *trnH-psbA* regions from *Blumea oxyodonta* (NCBI accessions EU195665 and BK013128) were served as the references when the Map to Reference tool was applied. Two assembling tools generated identical sequences in all of our samples.

The initial sequencing results showed no ambiguous base in the newly generated *Blumea* ITS sequences. Therefore, no cloning was conducted.

Sequence alignments for the studied regions were carried out applying Clustal W (Larkin et al. 2007) implemented in Geneious Prime 2022.1 and then visualized with Mesquite 3.7 (Maddison and Maddison 2021). As the earlier studies focused on *Blumea* plants (Pornpongrungrueng et al. 2007, 2009; Zhang et al. 2019), we then concatenated the alignments of ITS, *trnL-trnF*, *trnH-psbA* regions for the following analyses using Mesquite 3.7.

Many studies have suggested that taxa with extensive missing data would mislead the topology (e.g., Wiens 1998; Lemmon et al. 2009; Simmons 2012). Therefore, only the taxa with both ITS region and at least one chloroplast region will be applied in this study.

The best-fit nucleotide substitution model for the concatenated alignment was estimated with jModeltest 2.1.10 (Darriba et al. 2012). Both the maximum likelihood (ML) and Bayesian inference (BI) algorithms were then performed to infer the phylogenetic relationships among the studied *Blumea* taxa. The ML tree was reconstructed using RAxML-VI-HPC (Stamatakis 2006) in Geneious Prime 2022.1 with 1000 rapid bootstrap procedures and the best-fit nucleotide substitution model. The BI tree was generated utilizing MrBayes 3.2.6 (Huelsenbeck and Ronquist 2001; Ronquist et al. 2012) available in the CIPRES Science Gateway 3.3 (Miller et al. 2010) with the best-fit nucleotide substitution model and two independent Markov Chain Monte Carlo (MCMC) runs. A total of 5 × 10^6^ generations were executed in each MCMC run, and trees were sampled every 1000 generations. The posterior probabilities (pp) on the consensus BI tree were given by summing the last 75% of trees (burninfrac = 0.25). Above all, the ML and BI trees were drawn using FigTree 1.4.4 (Rambaut 2018).

## Results and discussions

### Phylogenetic analyses and insights

**Fig. 3:**
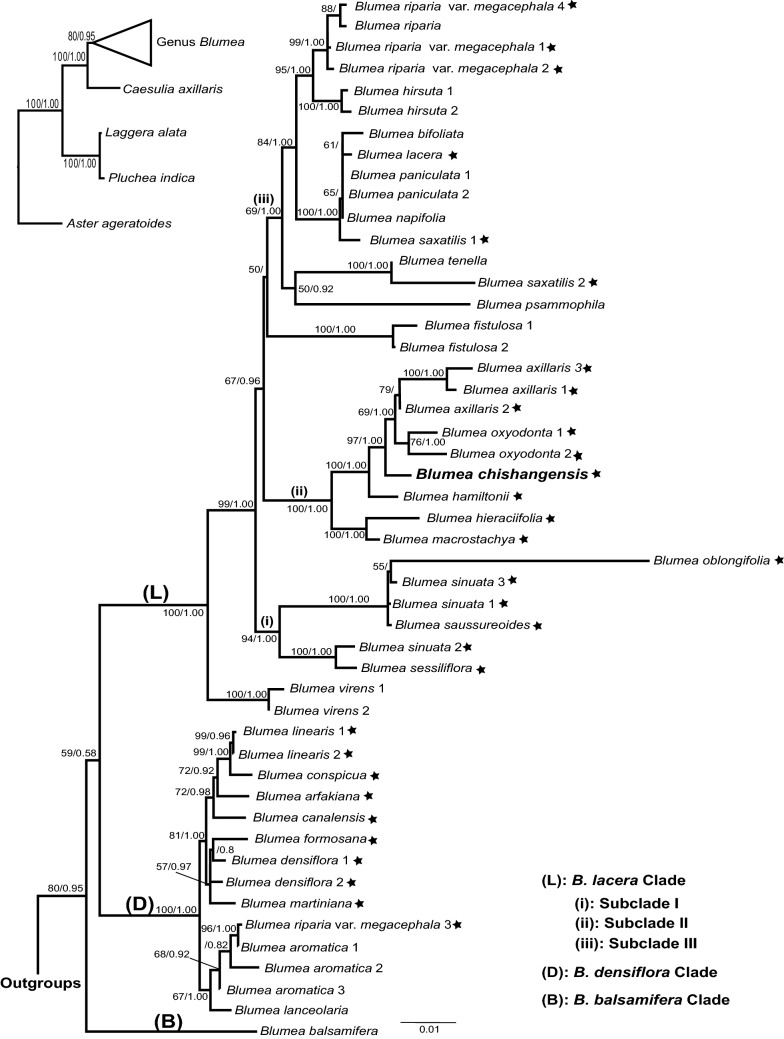
Phylogenetic tree of 31 *Blumea* taxa based on ITS, *trnL-trnF*, and *trnH-psbA* regions using the maximum likelihood estimations. Bootstrap values (bs)/posterior probabilities (pp) are shown at the internal nodes if bs is greater than 50 or pp is greater than 0.70. The clades are noted by following Pornpongrungrueng et al. (2007, 2009). *Blumea chishangensis*, the newly described species in the present study, are highlighted in bold. The taxa denoted with stars are discussed in the text. The number after taxa refer to Additional file 1

In total, 140 *Blumea* sequences––including 39 newly generated and 101 downloaded sequences––representing 32 *Blumea* taxa and 12 sequences representing four closely related outgroups were investigated in the present study (Additional file 1). Note that these numbers do not include the other six *Blumea* taxa which had been dismissed from the following analyses because these six taxa have only ITS region or only one chloroplast region (Additional file 1). The ML and BI trees were reconstructed based on the combined alignment (ITS, *trnL-trnF*, and *trnH-psbA* regions) with a length of 2054-bp. The maximum parsimony statistics and evolutionary model are provided in Table 2. Both ML and BI trees show the same topology (Additional file 2) and hence only ML is shown here (Fig. 3).

**Table 2:** Characteristics, maximum parsimony statistics, and evolutionary model of the studied alignment

DNA alignment	Number of outgroups/*Blumea* accessions	Length (bp)	Evolutionary model	Number of parsimony-informative sites [%]	CI/RI/RC/HI
All combined (ITS + *trnL-trnF* + *trnH-psbA*)	4/49	2054 (667 + 856 + 531)	GTR + I + G	385 (282 + 36 + 67) [18.74% (42.28% + 4.21% + 12.62%)]	0.704/0.848/0.597/0.296

*CI* consistency index; *RI* retention index; *RC* rescaled consistency index; *HI* homoplasy index

Our phylogeny (Fig. 3) supports Pornpongrungrueng et al. (2007, 2009)’s works that *Blumea* taxa are clustered in three groups––*B. lacera* clade, *B. densiflora* clade, and *B. balsamifera* clade. The relationships among the three clades remain unclear. Within the *B. lacera* clade, our data support Zhang et al. (2019)’s work that three subclades and two single species are recognized (Fig. 3).

The phylogenetic positions of five *Blumea* taxa (*B. chishangensis*, *B. conspicua*, *B. linearis*, *B. sinuata*, and *B. formosana*) are discussed in the following paragraphs, and the evolutionary information of the former three are firstly provided in this study.

Our results suggest that the new species, *B. chishangensis*, described here is a member of the subclade II in *B. lacera* clade (Fig. 3). This species is closely related to *B. oxyodonta* and *B. axillaris*, and all three species together are the sister group of *B. hamiltonii*. Interestingly, *B. axillaris*, *B. hamiltonii*, and *B. oxyodonta* are widely distributed, while *B. chishangensis* is one of the endemic *Blumea* taxa in Taiwan and has a restricted distribution. *Blumea axillaris* is the most widespread *Blumea* taxon distributed in tropical Asia (including Taiwan), tropical Africa, and northern Australia. Both *B. hamiltonii* and *B. oxyodonta* occur in southwest China, Indochinese Peninsula, and the Indian subcontinent, but not in Taiwan (Merrill 1910; Randeria 1960; Leu and Peng 1998; Chen and Anderberg 2011; Pornpongrungrueng et al. 2016).

*Blumea chishangensis* is morphologically similar to *B. hieraciifolia* (see the following discussion), and both species are members of subclade II. However, *B. chishangensis* and *B. hieraciifolia* had different evolutionary histories. The former is grouped with *B. oxyodonta* and *B. axillaris* while the latter shares the same ancestor with *B. macrostachya* (Fig. 3). The conflicts between molecular and morphological data are not rare in plants (e.g., Bremer and Struwe 1992; Schuster et al. 2018; Lu et al. 2021). These conflicts might infer some convergent evolution and/or hybridization in the evolutionary history (e.g., Ran et al. 2018; Soreng and Davis 2000; Liu et al. 2020).

*Blumea conspicua* occurs only on Ryukyu arc islands, Japan, Orchid Island, and Taiwan, and *B. linearis* is endemic to Orchid Island and Taiwan (Hayata 1911; Leu and Peng 1998; Peng and Leu 1999; Chen and Anderberg 2011). Our phylogeny points out that *B. conspicua* and *B. linearis* are sister taxa, and both of them belong to the *B. densiflora* clade (Fig. 3). These two species are closely related to *B. canalensis* and *B. arfakiana*, which distributions are also limited to some islands in the West Pacific Rim (Martelli 1883; Moore 1921). Additional samplings and/or DNA regions will be required to fully understand the diversification of this West Pacific Rim clade (e.g., Petersen and Hughes 2007; Chiang et al. 2014; Shaw et al. 2015).

With three samples, our phylogeny suggests that *B. sinuata* belongs to the *B. lacera* clade, but it is not a monophyletic taxon (Fig. 3). One *B. sinuata* sample is grouped with *B. oblongifolia* and *B. saussureoides* [pp = 1.00; bootstrapping value (bs) = 100], while another sample is sister to *B. sessiliflora* (pp = 1.00; bs = 100). Moreover, our data indicates that *B. formosana* belongs to the *B. densiflora* clade and it is clustered with *B. densiflora*, *B. martiniana*, and the West Pacific Rim *Blumea* clade (Fig. 3). However, Zhang et al. (2019)’s study suggested that *B. formosana* was grouped in the subclade III in *B. lacera* clade and sister to *B. saxatilis*, which had been asserted as a paraphyletic taxon in Pornpongrungrueng et al. (2009)’s study. Integrating Zhang et al. (2019)’s work and our work, it is likely that *B. formosana* is also a paraphyletic taxon. In fact, Pornpongrungrueng et al. (2009) had recognized several paraphyletic *Blumea* taxa––including *B. lacera*, *B. axillaris*, *B. saxatilis*, and *B. riparia* var. *megacephala*. Here, we report two more paraphyletic *Blumea* taxa, *B. formosana* and *B. sinuata*. All of the six paraphyletic taxa are relatively widespread. There might be some cryptic diversifications or taxa within a widely distributed taxon (e.g., Dick et al. 2003; Gottlieb 2004). Hybridization, introgression, and gene exchange with local taxa might also be the potential mechanisms that result in a widespread, but paraphyletic, taxon (e.g., Liu et al. 2018; Wen et al. 2020). Further taxonomic and molecular works will be demanded to provide a comprehensive classification and better understanding of the evolutionary history of *Blumea.*

### Morphological investigation on the new species

Detailed comparisons showed that this unknown taxon is morphologically similar to *B. hieraciifolia*, which is distributed to southeastern Asia and China (Leu and Peng 1998; Pornpongrungrueng et al. 2016), but still distinct in following features: leaves basal rosette or sub-basal rosette and a few cauline (vs. mostly cauline), leaf blade sparsely pilose or glabrescent (vs. silky villous), and capitula pedicelled (vs. capitula sessile). Additionally, *B. oxyodonta*, *B. axillaris*, and *B. hamiltonii* are included in the *B. lacera* clade (Pornpongrungrueng et al. 2007, 2009), also share similar gross morphology with the unknown taxon. However, *B. oxyodonta* can be clearly distinguished by its procumbent habit, spinous-toothed leaves, and mostly abortive anthers; *B. axillaris* differs in having purple flowers, distinctly petiolate leaves; *B. hamiltonii* is also distinct by having tomentose to wooly stem and leaf surface with glandular hairs and branch-tailed anthers bases. As the morphological characteristics of the unknown Taiwanese population could not match well with any described *Blumea* taxa, we consider it as a new species and propose the name “*B. chishangensis*” (see “Taxonomic treatment” section below). A key to *Blumea* species in Taiwan and the allies of *B. chishangensis* is also provided.

## Taxonomic treatment

*Blumea chishangensis* S. W. Chung, Z. H. Chen, S. H. Liu & W. J. Huang sp. nov. (Fig. 1, Fig. 2).

池上艾納香.

### Diagnosis

*Blumea chishangensis* is closely allied to *B. hieraciifolia* in morphological appearance but not in the evolutionary relationship (Fig. 3). The new species could be distinguished by the following features: sparsely pilose or glabrescent (vs. silky villous) leaves, the leaves margins regularly remote mucronulate (vs. double serrate or dentate), capitula with pedicelled (vs. capitula sessile or subsessile), and leaves basal rosette, sub-basal rosette or sometimes cauline (vs. mostly cauline).

### Type

TAIWAN. Taitung County: Chishang Township, Fuxing wetland, 250 m, 26 September 2019, *S.W. Chung 14025* (holotype TAIF!).

### Description

Perennial herb, erect, 30–60 cm high, with a fibrous rootstock. Stems simple, purple, usually unbranched, terete, 2–4 mm in diameter, puberulous or sometimes glabrate on basal part; leaves basal rosette or sub-basal rosette and a few cauline. Lower leaves larger, coriaceous, (5.5–)10–17.9 cm long, (1.2–)2–2.8 cm wide, spathulate to obovate-oblong, obtuse or acute at apex, the base attenuate into winged petiole, the margins regularly remote mucronulate, the upper surface often rugose and glabrescent when mature, the lower surface puberulous or glabrescent, veins 6–8 pairs. Capitula dense to lax panicles, pedunculate. Involucres campanulate-globose, with the phyllaries 3–4 series; the outer ones shorter, linear-lanceolate, 2–3 mm long, 0.7–0.9 mm wide, densely soft-villous on the dorsal surface with multicellular hairs and glands; the inner ones linear, 6–7 mm long, 0.5–0.6 mm wide, the margins and the apices ciliate. Receptacle convex, 2–4 mm in diameter, alveolate, glabrous. Corollas yellow, tubular; central florets 7–8 mm long, with 5 broadly triangular lobes, the lobes pubescent with multicellular hairs; outer florets filiform, 6–7 mm long, 2–3 lobed. Anther apical appendages truncate, bases with unbranched tails. Achene pale brown, oblong-terete, 1–1.2 mm long, ribbed, sparsely pubescent. Pappus white, caducous, 4–5 mm long.

### Distribution and habitat

*Blumea chishangensis* is endemic to Taiwan, recorded around the south and southeast region of Taiwan at elevations of 250–300 m. Plants grow on open grassland along the trail.

### Phenology

The flowering of *Blumea chishangensis* is observed from August to October and fruiting from September to November.

### Etymology

The epithet “chishangensis” refers to the name of the Chishang Township, Taitung County, Taiwan, where the novel species was discovered. Vernacular names, “池上艾納香” and “Chishang *Blumea*”, are also proposed.

### Conservation status

Endemic. Known from two restricted locations and one was only recorded once by Henry (1896) in Kaohsiung City over a hundred years ago. The present existing population would be negatively affected by an increase in human activities. AOO is less than 10 km^2^, and 100 individuals at most. We, therefore, evaluated it as Vulnerable [CR C2a] based on the latest IUCN guidelines (IUCN Standards and Petitions Committee 2022).

### Additional specimens examined (paratypes)

TAIWAN. Taitung County: Chishang Township, Fuxing wetland, 2 August 2019, *Z.H.Chen1847* (TAIF!). Kaohsiung City: Takow, *Henry 1942* (TAI-110021!).

Key to *Blumea* species in Taiwan. (Include all studied taxa in the Subclade II of *B. lacera* Clade; Taiwan’s species are highlighted in bold).

1. Pl﻿ants scandent; heads 10–15 mm across……………***B. riparia* var. *megacephala***.

1. Plants erect herbs or subshrubs; heads 3–6 mm across……………………….…….2.

2. Leaves nearly glabrous on both surfaces……………………………. ***B. lanceolaria***.

2. Leaves pubescent or glabrescent when mature………………………….………….3.

3. Petiole with appendages, pappus reddish………………………….. ***B. balsamifera***.

3. Petiole without appendages, pappus white…………………….…………….…….4.

4. Leaves distinctly petiolate; lobes of disc corolla purplish or pink ……….. ***B. axillaris***.

4. Leaves sessile or subsessile; corollas yellow or rarely pinkish…………………….5.

5. Involucres glandular hairy only, involucral bracts recurved at tips……***B. aromatica***.

5. Involucres both glandular hairy and villous; involucral bracts appressed or ascending………………………………………………….……………………….6.

6. Plants 1.5–3 m tall; leaves 30–45 cm long………………………..………………….7.

6. Plants less than 1.5 m tall; leaves less than 25 cm long…………………………….8.

7. Leaves linear or linear-lanceolate, 2.5-3.5 cm wide; corolla lobes of central florets with sessile glands and sparse multicellular hairs……..…….…………....***B. linearis***.

7. Leaves obovate or lanceolate, 10-15 cm wide, corolla lobes of central florets without multi-cellular hairs…………………………………..…….…..***B. conspicua***.

8. Plant prostate or procumbent, leaves spinous-toothed.….…..………..*B. oxyodonta*.

8. Plant erect, leaves not spinous-toothed…………………………………………….9.

9. Inflorescence a loose panicle………………………………………………………10.

9. Inflorescence a dense panicle or a terminal globose cluster.…………………..….13.

10. Leaf base acuminate, margin serrulate…………………………………***B. fomosana***.

10. Leaf base acute, margin coarsely dentate…………………………………………11.

11. Leaves irregularly laciniate or lyrate at base…………………….………….……12.

11. Leaves remotely serrate, not lobed…………………….……………..***B. oblongifolia***.

12. Leaves lyrate, usually pinnatipartite at base; receptacles pubescent, pilose, rarely glabrous…………………………………………………………..……..***B. sinuate***.

12. Leaves obovate, slightly lyrate; receptacles glabrous…………………….***B. lacera***.

13. Anther tails branched……………………………………….……..…. *B. hamiltonii*.

13. Anther tails unbranched………………………………………..………..………14.

14. Capitula 3–4 mm diam.; cypselas 5-ribbed………………………*B. macrostachya*.

14. Capitula 6–10 mm diam.; cypselas 10-ribbed………………………..…………15.

15. Leaves basal rosette or sub-basal rosette and a few cauline, sparsely pilose or glabrescent when mature, the margins regularly remote mucronulate; capitula pedicelled…....***B. chishangensis***.

15. Leaves mostly cauline, silky villous, margins double serrate or dentate, capitula sessile………………………………………………………….…***B. hieraciifolia***.

## Supplementary Information

**Additional file 1: Table S1.** List of the studied *Blumea *taxa, their GenBank accession numbers, and voucher information.

**Additional file 2: **Alignments and trees of the phylogeny of genus *Blumea*.

## Data Availability

All data generated and analyzed during this study are included in this published article and its supplementary information files.

## Abbreviations

BI: Bayesian inference
bs: Bootstrapping value
HAST: Academia Sinica, Herbarium
IBK: Guangxi Institute of Botany, Herbarium
IBSC: South China Botanical Garden, Herbarium
K: Royal Botanic Gardens, Herbarium
KUN: Kunming Institute of Botany, Chinese Academy of Sciences, Herbarium
ML: Maximum likelihood
P: Muséum National d’Histoire Naturelle, Herbarium
PE: Institute of Botany, Chinese Academy of Sciences, Herbarium
pp: Posterior probability
sp. nov.: Species nova (new species)
TAI: National Taiwan University, Herbarium
TAIF: Taiwan Forestry Research Institute, Herbarium
