# Supplementary material for: Blumea chishangensis sp. nov. (Asteraceae: Inuleae) from Taiwan and new insights into the phylogeny of Blumea
Source: Bot Stud. 2022 Jul 12;63:21. doi: 10.1186/s40529-022-00350-z (PMC9276887; doi:10.1186/s40529-022-00350-z)
Supplement: Supplementary file 1 — Additional file 1: Table S1. List of the studied Blumea taxa, their GenBank accession numbers, and voucher information. [file 40529_2022_350_MOESM1_ESM.docx]

**Additional file 1.** *Blumea* and outgroup taxa, the GenBank accession numbers, and voucher information of newly collected samples applied in the present study. The sequences indicated with asterisks are newly generated. Missing data are indicated with the em dash.

| **Taxon** | **ITS** | ***trnL-trnF*** | ***psbA-trnH*** | **Collector, Collection #** |
| --- | --- | --- | --- | --- |
| **Outgroups** |  |  |  |  |
| *Aster ageratoides* Turcz. | MH711534 | MW813970 | MW813970 |  |
| *Caesulia axillaris* Roxb. | EU195642 | EU195603 | EF210997 |  |
| *Laggera alata* (D. Don) Sch. Bip. ex Oliv. | FJ980335 | EF211025 | EF210979 |  |
| *Pluchea indica* (L.) Less. | LC194195 | MG452144 | MG452144 |  |
| **Ingroups** |  |  |  |  |
| *Blumea arfakiana* Martelli | EF210940 | EF211035 | EF210989 |  |
| *Blumea aromatica* DC. 1 | *OK104022 | *OK181928 | *OK120857 | Shih-Wen Chung 14281 |
| *Blumea aromatica* DC. 2  *Blumea aromatica* DC. 3 | EU195644 EF210950 | GU931327 KP052673 | EU195586 EF210998 |  |
| *Blumea axillaris* (Lam.) DC. 1 | *OK104028 | *OK181919 | *OK120850 | Shih-Wen Chung 14214 |
| *Blumea axillaris* (Lam.) DC. 2 | *OK104023 | *OK181929 | *OK120858 | Shih-Wen Chung 14236 |
| *Blumea axillaris* (Lam.) DC. 3 | KP052666 | GU931335 | EF210991 |  |
| *Blumea balsamifera* (L.) DC. | KF443294 | FM997837 | KP095162 |  |
| *Blumea bifoliata* (L.) DC. | EU195647 | EU195608 | -- |  |
| *Blumea canalensis* S.Moore | EF210967 | EF211062 | -- |  |
| *Blumea chishangensis* S.W.Chung, Z.H.Chen, S.H.Liu & W.J.Huang | *OK104025 | *OK181915; *OK181916 | *OK120847 | Shih-Wen Chung 14025 |
| *Blumea hirsuta* (Less.) M. R. Almeida 1  *Blumea hirsuta* (Less.) M. R. Almeida 2 | EU195648  EF210974 | EU195609  EF211069 | EU195587  EF211020 |  |
| *Blumea conspicua* Hayata | *OK104024 | *OK181930 | *OK120859 | Shih-Wen Chung 14846 |
| *Blumea densiflora* DC. 1  *Blumea densiflora* DC. 2 | EU195652 EU195650 | EU195613 EF211029 | EU195588 EF210983 |  |
| *Blumea fistulosa* (Roxb.) Kurz 1  *Blumea fistulosa* (Roxb.) Kurz 2 | EU195653 EF210935 | EF211030 GU931331 | EU195589 EF210984 |  |
| *Blumea formosana* Kitam. | *OK104032 | *OK181924 | *OK120854 | Wei-Jie Huang 1674 |
| *Blumea hamiltonii* DC. | EF210972 | EF211067 | EF211018 |  |
| *Blumea hieraciifolia* (D. Don) DC. | KP052662 | KP052679 | EF210985 |  |
| *Blumea lacera* (Burm. f.) DC. | EF210938 | EU195619 | EF210987 |  |
| *Blumea lanceolaria* (Roxb.) Druce | *OK104026 | *OK181917 | *OK120848 | Shih-Wen Chung 14219 |
| *Blumea linearis* C.-I Peng & W.P. Leu 1 | *OK104030 | *OK181922 | *OK120852 | Shih-Wen Chung 14218 |
| *Blumea linearis* C.-I Peng & W.P. Leu 2 | *OK104027 | *OK181918 | *OK120849 | Shih-Wen Chung 14219 |
| *Blumea macrostachya* DC. | EF210937 | EF211032 | EF210986 |  |
| *Blumea martiniana* Vaniot | EU195657 | EU195622 | EU195592 |  |
| *Blumea napifolia* DC. | EF210959 | EU195629 | EF211007 |  |
| *Blumea oblongifolia* Kitam. | KP052667 | GU931336 | -- |  |
| *Blumea oxyodonta* DC. 1  *Blumea oxyodonta* DC. 2 | EU195665  EF210956 | EU195630 EF211051 | EF211004 EU195598 |  |
| *Blumea paniculata* (Wall.) M.R. Almeida 1  *Blumea paniculata* (Wall.) M.R. Almeida 2 | EU195666  EF210941 | EU195631  EF211036 | EF210990  EU195599 |  |
| *Blumea psammophila* Dunlop | EF210943 | EF211038 | EF210992 |  |
| *Blumea riparia* (Blume) DC. | EU195667 | KP052685 | EF210993 |  |
| *Blumea riparia* var*. megacephala* Randeria 1 | *OK104029 | *OK181921; *OK181920 | *OK120851 | Shih-Wen Chung 14096 |
| *Blumea riparia* var*. megacephala* Randeria 2 | *OK104021 | *OK181926; *OK181927 | *OK120856 | Shih-Wen Chung 14167 |
| *Blumea riparia* var*. megacephala* Randeria 3 | MH674417 | -- | MH674426 |  |
| *Blumea riparia* var*. megacephala* Randeria 4 | KP052670 | GU931334 | EF211010 |  |
| *Blumea saussureoides* C.C. Chang & Y.Q. Tseng | KP052669 | GU931339 | -- |  |
| *Blumea saxatilis* Zoll. 1 | EF210946 | -- | EF210994 |  |
| *Blumea saxatilis* Zoll. 2 | EU195668 | EU195635 | -- |  |
| *Blumea sessiliflora* Decne. | EF210947 | EF211042 | EF210995 |  |
| *Blumea sinuata* (Lour.) Merr. 1 | *OK104033 | *OK181925 | *OK120855 | Shih-Wen Chung 14509 |
| *Blumea sinuata* (Lour.) Merr. 2 | *OK104031 | *OK181923 | *OK120853 | Shih-Wen Chung 14230 |
| *Blumea sinuata* (Lour.) Merr. 3 | EF210948 | EU195637 | EF210996 |  |
| *Blumea tenella* DC. | EU195671 | AF452500 | BK013129 |  |
| *Blumea virens* DC. 1  *Blumea virens* DC. 2 | EU195673 EF210957 | EU195640 EF211052 | EF211005 EU195602 |  |

Note that six *Blumea* taxa (*B. amakidophora, B. brevipes, B. flava, B. macrophylla, B. procera,* and *B. sylvatica*) were dismissed from this study because they have only ITS region or only one chloroplast region.
